# A novel hairpin library-based approach to identify NBS–LRR genes required for effector-triggered hypersensitive response in *Nicotiana benthamiana*

**DOI:** 10.1186/s13007-017-0181-7

**Published:** 2017-04-28

**Authors:** Cyril Brendolise, Mirco Montefiori, Romain Dinis, Nemo Peeters, Roy D. Storey, Erik H. Rikkerink

**Affiliations:** 1 Mt Albert Research Centre, The New Zealand Institute for Plant and Food Research Limited (PFR), 120 Mt Albert Road, Auckland, 1142 New Zealand; 2 INRA, Laboratoire des Interactions Plantes Micro-Organismes (LIPM), UMR441, CS52627, Chemin de Borde Rouge, 31326 Castanet-Tolosan, France; 3 Te Puke Research Centre, The New Zealand Institute for Plant and Food Research Limited (PFR), 412 No. 1 Road, RD 2, Te Puke, 3182 New Zealand

**Keywords:** Plant/pathogen interaction, Resistance gene, Hypersensitive response (HR), Effector screening, Multiple gene silencing

## Abstract

**Background:**

PTI and ETI are the two major defence mechanisms in plants. ETI is triggered by the detection of pathogen effectors, or their activity, in the plant cell and most of the time involves internal receptors known as resistance (R) genes. An increasing number of R genes responsible for recognition of specific effectors have been characterised over the years; however, methods to identify R genes are often challenging and cannot always be translated to crop plants.

**Results:**

We present a novel method to identify R genes responsible for the recognition of specific effectors that trigger a hypersensitive response (HR) in *Nicotiana benthamiana*. This method is based on the genome-wide identification of most of the potential R genes of *N. benthamiana* and a systematic silencing of these potential R genes in a simple transient expression assay. A hairpin-RNAi library was constructed covering 345 R gene candidates of *N. benthamiana*. This library was then validated using several previously described R genes. Our approach indeed confirmed that Prf, NRC2a/b and NRC3 are required for the HR that is mediated in *N. benthamiana* by Pto/avrPto (prf, NRC2a/b and NRC3) and by Cf4/avr4 (NRC2a/b and NRC3). We also confirmed that NRG1, in association with N, is required for the Tobacco Mosaic Virus (TMV)-mediated HR in *N. benthamiana*.

**Conclusion:**

We present a novel approach combining bioinformatics, multiple-gene silencing and transient expression assay screening to rapidly identify one-to-one relationships between pathogen effectors and host R genes in *N. benthamiana*. This approach allowed the identification of previously described R genes responsible for detection of avirulence determinants from *Pseudomonas*, *Cladosporium* and TMV, demonstrating that the method could be applied to any effectors/proteins originating from a broad range of plant pathogens that trigger an HR in *N. benthamiana*. Moreover, with the increasing availability of genome sequences from model and crop plants and pathogens, this approach could be implemented in other plants, accelerating the process of identification and characterization of novel resistance genes.

**Electronic supplementary material:**

The online version of this article (doi:10.1186/s13007-017-0181-7) contains supplementary material, which is available to authorized users.

## Background

In the course of evolution plants have developed various defence mechanisms to fight invading pathogens. The first level of plant immunity relies on the detection of pathogen-associated molecular patterns (PAMPs), such as flagellin, elongation factor EF-Tu or chitin in the apoplast of the plant cell by specific extracellular receptors (PRRs), and is referred to as PAMP-triggered immunity (PTI) also known as the basal or early immune response. Induction of PTI induces a signal cascade that leads to various plant defence mechanisms such as cell wall reinforcement by callose deposition and the production of antimicrobial chemicals including reactive oxygen species (ROS) or antimicrobial proteins and enzymes. PTI is usually sufficient to prevent the establishment of non-pathogenic microorganisms. However, pathogenic bacteria have gained the ability to inject virulence factors (effectors) in the plant cell to suppress PTI and restore favourable growth conditions for infection. In turn, plants have evolved a second layer of immunity mediated by the recognition of these effector(s) by largely internal receptors (R proteins) referred to as effector-triggered immunity (ETI). Upon recognition of the effector, these receptors ultimately induce a localised programmed cell death also called hypersensitive response (HR) to restrict proliferation of the pathogen. The most common class of these receptors acts inside the cell and typically contain nucleotide-binding site (NBS) and leucine-rich repeat domains (LRR). Co-evolution of plants and their pathogens in an arms race (to evade and then regain recognition of one another), illustrated by the zigzag model [1], leads to an increasing diversity of R proteins, effectors and mechanisms of resistance and susceptibility.

Many examples of gene-for-gene resistance have been described. Tomato cultivars expressing the R gene *Cf*-*9* were shown to be resistant to the fungus *Cladosporium* pathovars expressing the avr9 effector [2]. Interestingly, this resistance mechanism is transferable to transgenic tobacco expressing the tomato *Cf*-*9* gene [3]. Many microbial-plant interacting pairs of proteins have also been characterized, such as avrRPM1/RPM1 and avrRpt2/RPS2 in *Arabidopsis*, or avrPto/Pto in tomato [4–6]. Host recognition of the effector can occur through direct interaction between the effector and the R protein. This is the case for *Magnaporthe oryzae* effector Avr-Pia, which was shown to interact with the RGA5/RGA4 pair of R proteins [7] or the *Pseudomonas syringae* avrPto effector and tomato Pto decoy kinase [6]. In many cases, however, recognition is indirect and relies on the concept that some host targets are guarded by R proteins, which instead detect modifications by the effector of the target’s integrity or function to activate ETI. The RIN4 protein in *Arabidopsis* is a well-documented example of a host protein targeted by at least three different effectors of *Pseudomonas* (AvrRpm1, AvrRpt2 and AvrB) and guarded by at least two R proteins (RPM1 and RPS2) able to induce HR upon RIN4 modification [5, 8]. More recently, Saunders et al. [9] demonstrated that resistance to *Phytophthora infestans* in potato, mediated by the recognition of the AVR2 effector by the R protein R2, requires the presence of the host target BSL1 with which both AVR2 and R2 physically interact. Identification of the R proteins responsible for effector recognition in plants is a valuable source of information for breeding resistant cultivars, be this by traditional breeding or transgenic approaches. However, the durability of resistance associated with particular R proteins remains problematic as the extreme selective pressure they exert causes many to be overcome by modifications of the pathogen’s repertoire of effectors. Consequently, successful resistance breeding strategies rely on stacking resistance from different origins (gene pyramids) in the same cultivar to reduce the chances of a pathogen evading recognition mediated by a single R protein. Furthermore, these strategies could be used in association with gene rotation to further enhance durability [10].

Identification of new R genes remains a challenging task. Most of the R genes characterized to date have been identified through approaches that are often labour-intensive and not always applicable to crop plants. A comprehensive genetic toolkit in *Arabidopsis* has permitted the identification of many R genes in this model plant (RPM1 [11], RPS4 [12, 13], RPS2 [4], TAO1 [14]); however, crop plants do not always benefit from the same panel of genetic tools such as mutant collections or sequenced and well annotated genomes. Mapping approaches have successfully identified resistance markers directly in crop plants but usually required additional experiments to identify the actual associated gene. In the era of plant genomics, increasing numbers of plant and pathogen genomes have been sequenced and provide valuable resources. These resources can be used to develop molecular and biochemical approaches such as Yeast-two-Hybrid (Y2H) or co-immunoprecipitation to identify host targets of pathogen effectors (AVR-Pia/RGA5 [7]; Pto/avrPto [6]), and bioinformatics approaches for more high throughput and genome-wide studies of plant resistance such as the NB–LRR Resistance gene enrichment and sequencing-based method described recently [15–17]. The identification and cloning of resistance genes, however, remains a difficult task and to address this we present a novel approach for the identification of disease resistance genes in *N. benthamiana*. Taking advantage of the availability of the *N. benthamiana* genome (Boyce Thompson Institute for Plant Research—Genbank: PRJNA170566), sequences of most of the potential R genes of the *N. benthamiana* genome were identified based on the presence of an NBS domain and used to create an RNAi knock-out library. The experimental procedure described was designed and initially tested using the well-described tomato-*P. syringae* pv. *tomato* interaction system that involves the Pto decoy kinase, the avrPto effector and the Prf R protein, and which can be recreated in the related solanaceous host *N. benthamiana*. Subsequently, the screen of our knock-out library with the avrPto effector led to the identification of additional R genes previously shown as required for Pto/avrPto-triggered HR (NRC2a/b and NRC3). In addition, we also confirmed the requirement of the *NRC2a/b* and *NRC3* R genes for recognition of *Cladosporium* Cf-4/avr4 effector, and that the proteins N and NRG1 are required for TMV recognition in *N. benthamiana*, thereby demonstrating that this approach can be applied to a broad range of plant pathogens.

## Results and discussion

### Genome-wide identification of *Nicotiana benthamiana* R genes

**Fig. 1:**
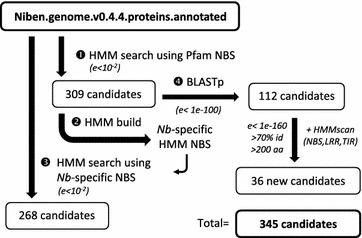
Schematics of the procedure to identify NBS domain-containing proteins in *N. benthamiana* annotated genome. HMMsearch of *N. benthamiana* annotated proteins using the standard Pfam NBS HMM led to the identification of 309 NBS-containing candidates. The 309 NBS domains were aligned using ClustalW and used to build a *N. benthamiana*-specific NBS HMM, which was subsequently used to screen again the *N. benthamiana* annotated proteins. However, no new candidates were identified in the 268 entries generated. *N. benthamiana* annotated proteins were further searched by BLASTp using the 309 candidates. New R gene candidates were further selected and screened for the presence of NBS, LRR and TIR domains using HMMscan

Predicted proteins from the annotated genome of *N. benthamiana* (Niben.genome.v0.4.4) [18] were screened for the presence of an NBS domain by similarity search using a Hidden Markov Model (HMM) corresponding to the generic NBS domain of the Pfam database (PF00931) (Fig. 1). A total of 309 protein sequences were selected. The NBS domains of the 309 candidates were aligned using CLUSTALW and used to generate an *N. benthamiana*-specific NBS HMM to be used in a second round screen of the *N. benthamiana* predicted proteins. However no additional NBS-containing proteins were identified. The *N. benthamiana* genome was then searched for additional R gene candidates using the initial 309 candidates to perform a batch BLASTP search against *N. benthamiana* predicted proteins. A high quality subset (E-value <1e−100) of 112 additional candidates was selected and screened for the presence of NBS, LRR and Toll-Interleukin Receptor (TIR) domains using HMMscan. Candidates containing either a partial NBS domain, a TIR domain or an LRR domain were retained. Most of the candidates containing domains similar to ABC transporter, ATPase domain and other P-loop related domains were excluded by increasing the selection stringency (E-value <1e−160, identity to query >70% and minimum sequence length of 200 residues). Results were further curated manually by visual analysis of the BLASTP alignments and corresponding gene models in JBrowse (https://solgenomics.net/organism/Nicotiana_benthamiana/genome) to eliminate potential partial protein due to mis-annotation. As a result, 36 additional candidates were retained for a total of 345 R gene candidates. This number is comparable with the number of R genes previously identified in the related species *Nicotiana tabacum* (281 R genes) [19]. More recently, 233 NBS–LRR proteins were identified in *N. benthamiana* [20], which are all included in our 345 protein set. This difference in *N. benthamiana* gene number could partially be due to the different protein prediction sets used in the two studies (v0.42 vs. v0.44). Additionally, because we intended to use the identified R gene sequences in a systematic silencing approach, our selection strategy erred on the side of inclusiveness. This strategy allowed us to identify any partial genes that may have an activity but may also include partial sequences originating from a same gene due to inaccuracies of the genome annotation, which may have led to an over-estimation of the number of functional candidates.

### Hairpin library design

**Fig. 2:**
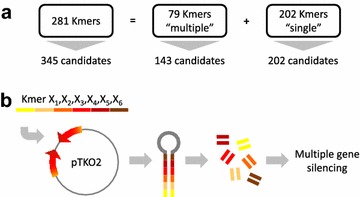
Hairpin library design. **a** A total of 281 Kmers were designed, with 202 Kmers targeting a unique gene of *N. benthamiana* annotated genome with 100% identity, and 79 kmers targeting two or more genes. **b** To produce multiple gene silencing constructs, groups of six kmers were synthesized in tandem (4 “singles” and 2 “multiple”), cloned in a Gateway-enabled pUC57 vector and eventually recombined by LR reaction into the hairpin-producing destination vector pTKO2

**Fig. 3:**
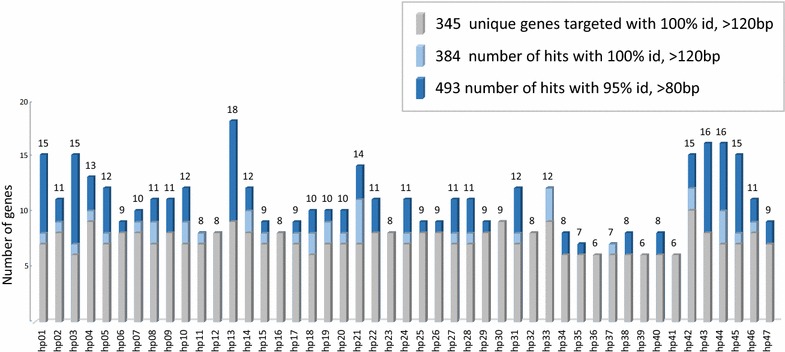
Distribution of the number of genes targeted by each hairpin construct. Three hundred and forty-five unique genes are targeted by the 47 constructs of the hairpin library (*grey*). When including redundancies, 384 genes are targeted (*light blue*) with 100% identity over the full length of each kmer. This number goes up to 493 genes with 95% identity to each kmer and a minimum length of 80 bp (*dark blue*), representing a redundancy of about 43%

A set of DNA fragments (kmers) was designed from the 345 candidate coding regions and filtered based on gene coverage of the whole set of the potential candidates using a Perl script. A total of 281 kmers of 120–150 bp with 100% identity to their targets were obtained to cover the 345 candidate genes. Of these, 79 kmers of 150 bp length target multiple R gene candidates (143 unique genes) and 202 kmers of 120 bp target a single R gene candidate (202 unique genes) (Fig. 2). To increase the practicability of our screen by reducing the number of individual constructs of the library, we evaluated whether it was possible to down-regulate multiple unrelated genes by inserting multiple kmers in tandem in a single hairpin. The silencing efficiency of hairpins containing multiple fragments was evaluated in a pilot experiment in which two 150 bp kmers targeting two distinct reporter genes were inserted at the most distal and most proximal ends of the hairpin containing a total of four kmers. This preliminary experiment showed that both reporter genes (*NtAN1* and *RPS2*) were efficiently silenced (Additional file 1: Fig. S1). Therefore we hypothesised that intermediate positions within the fragment should also lead to a significant level of gene knockdown. Our supposition was confirmed by recent work of Czarnecki et al. [21] who showed that multiple unrelated gene knockdown can be achieved by a single synthetic RNAi fragment regardless of the position of the kmer. To generate our RNAi library, up to six kmers were synthesized in tandem and recombined into the hairpin-producing destination vector pTKO2 (Fig. 2b) using Gateway cloning (Invitrogen, USA). The resulting 47 hairpin constructs, each targeting from 6 to 11 different R gene candidates, were transformed into *Agrobacterium tumefaciens* and used for transient expression in *N. benthamiana*. As described above some of the kmers were designed to target multiple related R genes sharing enough DNA sequence identity. Conversely some R gene candidates were targeted more than once due to strong sequence similarities between different regions of the gene. We calculated that the whole library would produce 384 hits at 100% identity over the 345 candidates (Fig. 3) representing 11% redundancy. However, the actual level of knockdown redundancy is underestimated considering that some level of knockdown is likely to occur with a lower level of sequence identity over the length of the kmer. For example, with 95% identity over a length of minimum 80 bp, the level of redundancy would increase to approximately 43% (Fig. 3). Since the relative position of mismatches in these kmers may aslo affect their ability to silence, it is currently not possible to calculate the actual redundancy level.

### Experimental design

The principle of our approach was to identify the R gene responsible for the HR triggered by an effector of interest in *N. benthamiana* by silencing the corresponding R gene involved in the mechanism of recognition of the effector and monitoring the disappearance of the HR. Each individual hairpin construct of the library was transiently co-expressed sequentially with the effector of interest in *N. benthamiana* leaves and the resulting response was compared with the HR triggered by the effector alone. If one of the multiple R genes targeted by the hairpin was involved in the recognition of the effector, the HR should be abolished or at least reduced compared with the effector alone. Each kmer from the hairpin was then cloned and tested individually under the same conditions against the effector to identify which one was responsible for the reduced HR phenotype and the corresponding targeted R gene candidate(s) was (were) identified.

**Fig. 4:**
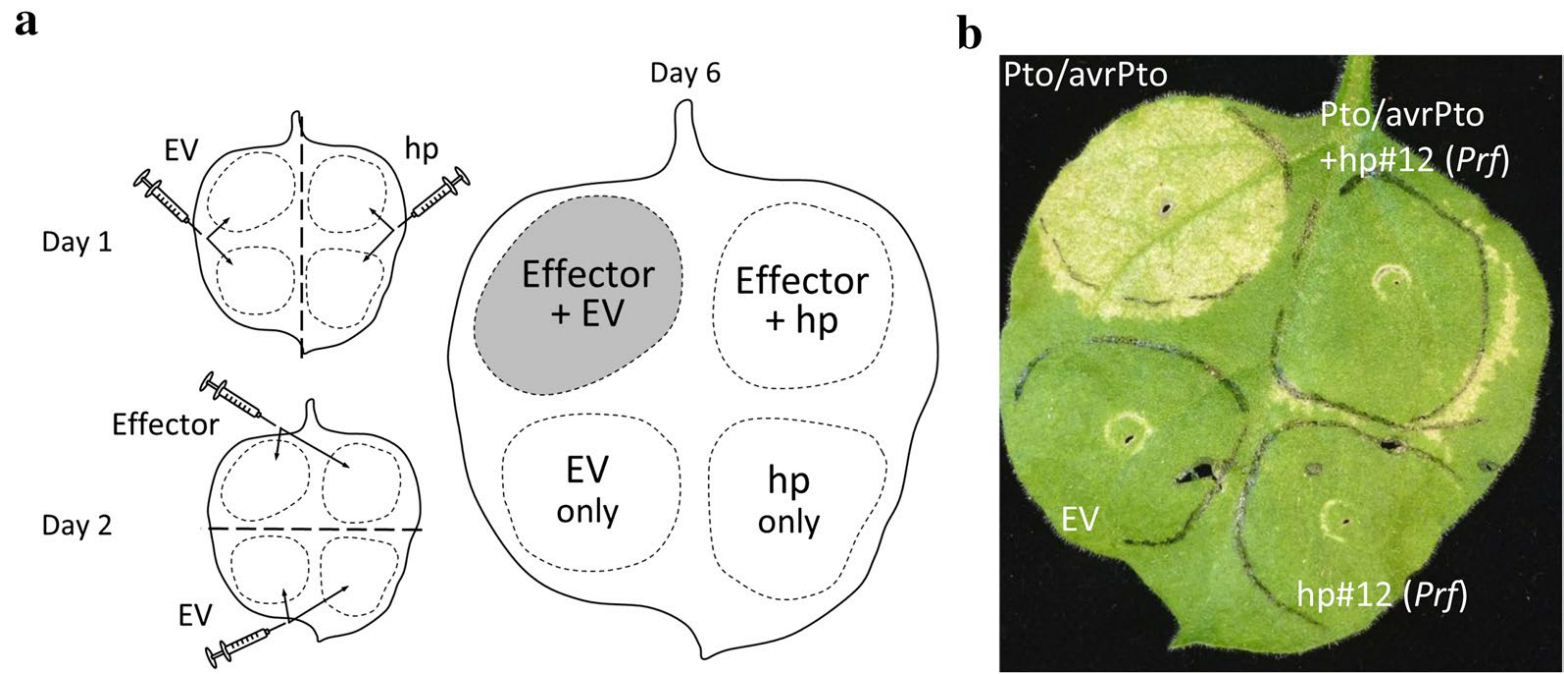
Pto/avrPto-triggered HR is cancelled by hp#12 pre-infiltration. **a** General design of the assay: *N. benthamiana* leaf was infiltrated with *Agrobacterium* containing the hairpin construct or corresponding empty vector (EV) in two distinct patches on the *right-hand side* and *left hand side* of the leaf respectively and infiltration areas were marked with a felt-tip pen. The following day, *Agrobacterium* containing the effector construct, or corresponding empty vector (EV), were infiltrated in the pre-infiltrated patches of the *top half* and *bottom half* of the leaf respectively. Development of the HR was monitored during the following 3–7 days depending on the strength of the HR. **b** *Agrobacterium* suspension (OD 0.2) carrying the hairpin hp#12 or corresponding empty vector was infiltrated in *N. benthamiana* leaf and the *Agrobacterium* suspension (OD 0.2) expressing the Pto/avrPto construct was infiltrated 24 h later on the pre-infiltrated patches as designed in (**a**). Photos was taken 4 days after Pto/avrPto infiltration

To optimize the experimental conditions we used the previously described patho-system Pto/avrPto and Prf. *Agrobacterium*-mediated transient co-expression of the avirulence protein avrPto and the kinase protein Pto causes HR in *N. benthamiana* leaves [6]. Moreover, avrPto recognition is dependent on the NBS–LRR protein Prf [22]. Hairpin hp#12 of our library contains a kmer targeting the two *N. benthamiana* homologs of the tomato *Prf* gene (NbS00001223g0007.1 and NbS00008803g0010.1). Hp#12 was pre-infiltrated in *N. benthamiana* leaves and constructs encoding both Pto and the avrPto effector were infiltrated 24 h later in the same leaf patch (Fig. 4a). The HR induced by Pto/avrPto expression was visible 3 days after infiltration but no HR developed when hp#12 was pre-infiltrated (Fig. 4b). Similar results were obtained when hp#12 was pre-infiltrated 48 h before Pto/avrPto (data not shown). However, co-infiltration of hp#12 and Pto/avrPto only led to a partial reduction of the HR (not shown) suggesting that the hairpin construct requires sufficient time to reduce/knockdown *Prf* expression, allowing sufficient discrimination to detect differences in the HR triggered by avrPto.

### Validation of the approach

**Fig. 5:**
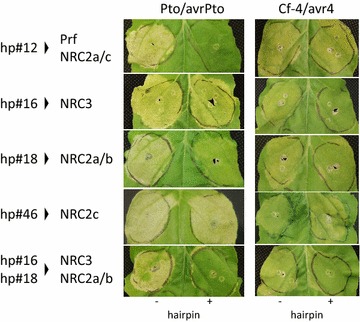
NRC2a/b and NRC3 are general regulator required for Pto/avrPto- and Cf-4/avr4-triggered HRs. The 47 constructs of the R gene knockout library were screened with Pto/avrPto effector as described in Fig. 4. Pre-infiltration of hp#16 and #18 was found to induce a slight reduction of the Pto/avrPto-triggered HR; however, combination of the two constructs completely cancelled the HR (*left panels*). Hp#16 and hp#18 were also found to reduce the Cf-4/avr4-triggered HR with a similar additive effect (*right panels*). Photos were taken 4 days after infiltration for Pto/avrPto and 7 days after infiltration for Cf-4/avr4

To validate our approach, Pto kinase and the avrPto effector were used to screen the 47 constructs of the library. Two additional hairpins hp#16 and hp#18 were identified that were able to reduce the Pto/avrPto-triggered HR (Fig. 5, left panel). Hp#16 induced only a marginal reduction of the HR whereas a more convincing reduction was obtained with hp#18. Interestingly, the combination of hp#16 and hp#18 had an additive effect, leading to a complete abolition of the HR. Sequence analysis revealed that hp#16 and hp#18 contain fragments u66 and m35 targeting the R genes *NRC3* and *NRC2a* and *2b* respectively, which were recently described as helper proteins required for Pto-mediated cell death [23]. The synergy effect obtained with hp#16 and hp#18 suggests some level of functional redundancy between NRC3 and NRC2a/b. Our screen also confirmed that the *NRC2c* gene, targeted by hp#46 was not required for Pto/avrPto-mediated HR as also described by the same authors [23]. It should be noted that in addition to *Prf* hp#12 also targets both and *NRC2a* and *NRC2c*. The result obtained with hp#18 supports the hypothesis that, in addition to *Prf*, the *NRC2a* silencing might partly contribute to the reduced HR obtained with hp#12. On the contrary, hp#46 result suggests that *NRC2c* silencing did not contribute to the reduction of the hp#12-induced HR.

To further validate the approach, hairpins #12, #16, #18 and #46 were also used on the fungal patho-system Cf-4/avr4 from *Cladosporium fulvum*, co-expression of which triggers an HR in *N. benthamiana* [24]. Hp#16 and hp#18, but not hp#12 nor hp#46, were able to reduce the Cf4/avr4-mediated HR (Fig. 5, right panel). Again hp#16 and hp#18 had an additive effect on cancelling the HR. These results are in agreement with research [23] showing that NRC2a/b and NRC3 were required for Cf-4/avr4-triggered HR in *N. benthamiana*. Hence these results extend the use of our library to identify R genes required for detection of both fungal and bacterial effectors.

**Fig. 6:**
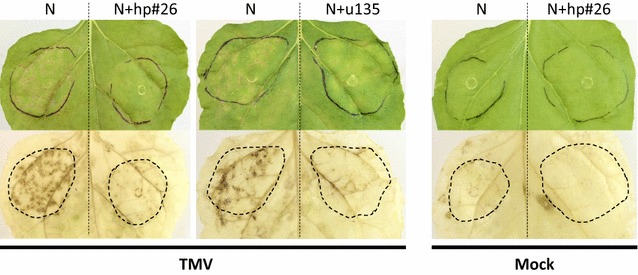
TMV-induced hypersensitive response (HR) in *N*-infiltrated leaves is cancelled by *NRG1* silencing. Hairpins hp#26 or u135 or the corresponding empty vector were co-infiltrated with *N* in *N. benthamiana* leaves and the infiltrated areas were marked with a felt-tip pen. TMV was inoculated 24 h after *Agrobacterium* infiltration. HR was visible from 4 days after TMV inoculation and photo were taken 6 days post-inoculation. H_2_O_2_ production in the same leaves was monitored using DAB staining

Finally, the *NRG1* gene, together with *N*, were previously shown to mediate resistance to Tobacco Mosaic Virus (TMV) in *N. benthamiana* [25]. The u135 fragment targeting the *NRG1* gene was identified in the library on hp#26 and used to assess the effect of *NRG1* silencing on the TMV resistance response in *N. benthamiana* leaves. *Agrobacterium* strains containing the hp#26 and a construct expressing the *N* gene from tobacco were co-infiltrated in *N. benthamiana* leaves as described above and TMV was inoculated 24 h or 48 h post-*Agrobacterium* infiltration. An HR was visible from 5 days post-TMV inoculation in the leaf patch expressing N but no HR was detected when N and hp#26 were co-expressed (Fig. 6). Identical results were obtained when the 120 bp u135 kmer was individually subcloned and tested. This results indicate that our R gene RNAi library approach is also able to identify R genes involved in resistance to viruses in *N. benthamiana*.

## Conclusion

We present a simple yet robust approach to identify R genes responsible for the recognition of effectors of interest in *N. benthamiana*. Our approach integrates two bioinformatics strategies (R gene identification and kmer design) with an efficient pooled-hairpin transient expression strategy. Three hundred and forty-five potential R gene candidates were identified in silico in the annotated *N. benthamiana* genome and a set of RNAi constructs was designed to specifically down-regulate each candidate. To reduce the size of the hairpin library to a manageable number of constructs, up to six fragments (kmers) targeting unrelated R genes were concatenated per RNAi construct, generating a hairpin library consisting of 47 RNAi constructs. Using a simple experimental design we tested the ability of our system to confirm that Prf mediates the recognition of avrPto in the presence of Pto. In the process we also confirmed that the NRC2 and NRC3 R genes are required for the Pto/avrPto-triggered HR as well as for the Cf-4/avr4-triggered HR, as was recently demonstrated [23]. In addition we showed that the TMV-triggered HR in *N. benthamiana* leaves transiently expressing N is cancelled in the presence of a hairpin targeting the *NRG1* R gene, demonstrating that the use of our library can also be extended to identify R genes required for resistance to viral pathogens.

The strategy we have developed here in *N. benthamiana* is potentially of much broader utility in crop plants, where the identification of resistance genes is still a significant barrier to progress in developing durable resistance strategies against pathogens. *Agrobacterium*-mediated transient gene expression has already been used successfully in many model and crop plants such as *Arabidopsis*, lettuce, tomato [26], tobacco [24], potato [27], wheat [28], or pea [29]. In addition, the cost of *de novo* genome sequencing of these crops to develop the data required for the bioinformatics components of our strategy is ever reducing as technology progresses. However, several important crop systems do not yet offer a transient expression system as simple and efficient as for *N. benthamiana*. Development of more efficient transient gene delivery/silencing systems in these hosts will be required. This may include the use of viral vectors, likely derived from viral species able to infect these hosts naturally but as yet poorly characterized in some crops. Transient expression of the library via viral vectors would present some advantages. One potential limitation of our approach is that the expression of the hairpin via *Agrobacterium* could trigger some level of plant innate immunity when applied to the leaf, which could interfere with further inoculation of the pre-infiltrated patch. Although it was not a problem in our case to express the effector of interest in the pre-infiltrated patch via *Agrobacterium*, we have found in preliminary experiments that *P. syringae* pv. *tomato* DC3000 infection was strongly inhibited by the *Agrobacterium* pre-infiltration (results not shown). This suggests that other means to deliver the effector of choice in pre-infiltrated leaves, for instance using the *Pseudomonas fluorescens* T3S-engineered strain [30], might be inefficient as well. Some of these barriers could be overcome by using viral vector-based gene delivery systems.

The identification of R genes and their corresponding effectors has long been an arduous task. We describe a new approach to rapidly identify R genes in *N. benthamiana* and we show how this powerful screening tool is able to identify resistance genes to several kind of plant pathogens including bacteria, fungi and viruses. This method could be used to identify R genes required for the recognition of effectors/elicitors originating from a much broader range of plant pathogens as long as they trigger a visible HR in *N. benthamiana* and could also be easily adapted to other plant hosts.

## Methods

### Bioinformatics

R gene identification: Predicted proteins of the *N. benthamiana* genome Niben.genome.v0.4.4 were screened using the HMMER 3.1b1 and the raw Hidden Markov Model (HMM) corresponding to the Pfam NBS (NB-ARC) domain (PF00931) as described previously [31, 32]. Default inclusion threshold of 0.01 was used (outfile provided in Additional file 2). *N. benthamiana*-specific NBS HMM was generated using the HMMbuild module using default parameters. Batch BLASTP of the 309 candidates on the *N. benthamiana* predicted proteins was performed with a maximum hits of 30 and a Max E-value of 1e−10 using the BLOSUM62 matrix. A high quality subset with an E-value <1e−100 was selected which included the 309 initial candidates and 112 new candidates. The 112 were screened for the presence of NBS, LRR and Toll-Interleukin Receptor (TIR) domains using HMMscan using default settings. The list of candidates was further curated by applying additional selection criteria: BLASTP E-value <1e−160, identity to query >70% over a sequence length of minimum 200 residues. The final list of candidates was manually checked for the presence of *N. benthamiana* homologs to known NBS-containing genes identified by BLASTP of the *N. benthamiana* predicted protein set (including RPM1, RPS2, RPS4, N, NRC1/2/3, NRG1, Prf, RRS1, ADR1 and RPW8). For all the genes tested, the five best hits obtained were present in the final 345 gene list. Conversely, the best hits retrieved using proteins containing more distant P-loop-related domains (such as ATPase, MAP kinase, ABC transporter, Receptor-like Kinase domains) were not present in the 345 gene list. Proteins tested included AtCRT1/AEE86644.1 (ATPase domain), AtMKK2/OAP00208.1 (MAP kinase domain), NOD1/O52618.3 (ABC transporter), EHD2/OAO99730.1 (P-loop and coiled-coil domains) and LRK1/AB247455.1 (LRR-RLK domain).

Kmer design: input FASTA files of the 345 DNA sequences were fragmented to produce all possible per gene DNA fragments (kmers) of 120 bp for subsequent filtering and picking. Filters used were designed to ensure a minimum hit rate of distinct genes was met and kmers that were duplicated within the same gene were removed. The final list of kmers were chosen with a set covering algorithm implemented in Algorithm::SetCovering (https://metacpan.org/pod/Algorithm::SetCovering and Additional file 3). Two hundred and eighty-one kmers were obtained, consisting of 202 ‘single’ kmers of 120 bp targeting a single R gene candidate and 79 ‘multiple’ kmers targeting two or more candidates. Sequence length of the ‘multiple’ kmers was arbitrarily extended to 150 bp in an effort to enhance the production of RNAi intended to silence multiple homologous genes. A list of the 345 gene candidates and corresponding kmers are provided in the Additional file 4: Table S1.

### Hairpin library and other constructs

Library construction: A total of 47 fragments consisting of 6 kmers manually arranged (4 ‘single’ and 2 ‘multiple’) and flanked by the Gateway attL1 and attL2 sequences were synthesized (GenScript) and cloned into the pUC57 vector, enabling each fragment to be cloned subsequently into the pTKO2 vector [33] by LR recombination following the manufacturer procedure (Invitrogen). The resulting 47 pTK02 constructs were transformed into *Agrobacterium* GV3101 (MP90). *35S:AVR4* and *35S:Cf*-*4* constructs and the Pto/avrPto construct have been described previously [24, 34]. The *N* gene genomic DNA sequence was amplified from *Nicotiana tabaccum* genomic DNA and cloned into the pHEX2 vector. To this end the pHEX2 vector was linearized using *Eco*R1 and *Hin*dIII and the *N* amplicon was inserted using the In-Fusion^®^ HD Cloning Kit from Clontech following the manufacturer instructions. The Kmer composition of the hairpins described in this study (#12, #16, #18, #26 and #46) is provided in the Additional file 4: Table S1. The individual fragment from hp#26 specifically targeting NRG1 (u135) was amplified from hp#26 and cloned using the In-Fusion^®^ HD Cloning Kit into the pENTR/SD/D-TOPO vector (Invitrogen) previously linearized by inverse PCR. The pENTR/SD/D-u135 entry clone was then recombined into the pTKO2 vector by LR recombination to generate the pTKO2-u135 hairpin construct (Additional files 5, 6).

### Plant material and transient assays in *N. benthamiana*

*Nicotiana benthamiana* plants were grown in 16 h-light/8 h-dark conditions at 22–24 °C until they reached the 6–8 leaves stage with the two youngest leaves big enough to be infiltrated. *Agrobacterium* infiltration into *N. benthamiana* was described previously [35]. In brief, *Agrobacterium* strain GV3101 containing the construct of interest was grown overnight at 28 °C in appropriate selective media, resuspended in infiltration buffer (10 mM MgCl_2_, 10 µM acetosyringone) and incubated for a minimum of 2 h before infiltration. Final concentration for the assay was adjusted to OD_600_ of 0.2 for the hairpin library constructs and the Pto/avrPto construct, 0.4 for the Cf-4 and avr4 constructs and 0.5 for N, hp#26 and hairpin u135 for the TMV assay. Hypersensitive response was visually monitored from 3 to 7 days after infiltration.

### Library screening procedure

For the Pto/avrPto screen, *Agrobacterium* suspensions containing each of the 47 constructs of the library were infiltrated in at least 6 leaves (2 leaves per plants) as per the design in Fig. 4a. Infiltrated areas were marked with a felt-tip pen and *Agrobacterium* containing the Pto/avrPto or corresponding empty vector were infiltrated in the pre-infiltrated areas 24 or 48 h later. Photos of representative leaves were taken 3 days after Pto/avrPto infiltration. The same procedure was used for avr4/Cf-4 and hairpins #12, #16, #18 and #46 with 48 h-preinfiltration and photos were taken 7 days after avr4/Cf-4 infiltration.

### TMV inoculation assay

*Agrobacterium* strains containing the hairpins hp#26 or u135 and the N construct, in combination with corresponding empty vectors, were mixed at a ratio of 1:1 (OD_600_ of 0.5) and co-infiltrated following the experimental design described in Fig. 4a. TMV was inoculated either 24 h or 48 h after *Agrobacterium* infiltration as described previously [36]. In brief, 400 mg of *N. benthamiana* leaf infected with TMV was crushed and resuspended in 4 mL of 50 mM phosphate buffer (pH7.4). Crude extract was diluted 10 times and supplemented with carborundum. The whole surface of each leaf was gently rubbed using 50 μL of the diluted extract. Visible HR symptoms were scored after 4 to 7 days. H_2_O_2_ production was visualized using DAB staining as described previously [36]. The experiment was repeated twice with both 24 and 48 h *Agrobacterium* pre-infiltration producing similar results.

## Additional files

**Additional file 1: Figure S1.** Multiple gene silencing proof-of-concept assay. A construct including a fragment of *NtAN1*, two DNA spacer sequences and a fragment of *RPS2* (each of 150 bp) and flanked by the Gateway attL1 and attL2 borders (A) was synthesized and recombined by LR reaction into the pTKO2 vector to produce the pTKO2-hp#test construct. (B) *Agrobacterium*-mediated transient expression of RPS2 triggers an HR in *N. benthamiana* leaf and RIN4 negatively regulates *RPS2* activation [37]. Here we show that when *RPS2* and the hp#test construct are co-infiltrated (ratio 1:1) the HR is severely reduced, demonstrating that the 150 bp-kmer at the last position of the construct efficiently mediates RPS2 silencing. (C) AcMYB110 is a positive regulator of the anthocyanin biosynthetic pathway in kiwifruit. It was shown previously that AcMYB110 requires the endogenous NtAN1 bHLH transcription factor to induce anthocyanin biosynthesis when expressed in tobacco leaf [38]. Here we show that when AcMYB110 and either the hp#test construct (2) or a specific NtAN1 hairpin construct (3) are co-infiltrated (ratio 1:1), no anthocyanin accumulates in the leaf demonstrating that in both cases the endogenous NtAN1 is efficiently silenced.

**Additional file 2.** Outfile of the HMMsearch of the *N. benthamiana* predicted proteins using PF000931 (NB-ARC).

**Additional file 3.** Algorithm::SetCovering, Algorithms to solve the “set covering problem”. Copyright 2003 by Mike Schilli m@perlmeister.com.

**Additional file 4: Table S1.** List of the sequences of the 345 R gene candidates (tab 1) and the 281 kmers (tab 2) of the RNAi library, and association between R gene candidates and Kmers (tab 3) and composition of the hairpin constructs used in this study (tab 4).

**Additional file 5.** Sequences of the 345 R gene candidates (FASTA format).

**Additional file 6.** Sequences of the 281 Kmers (FASTA format).

## Abbreviations

R genes: resistance genes
*N. benthamiana*: *Nicotiana benthamiana*
PAMPs: pathogen-associated molecular patterns
PTI: PAMP-triggered immunity
ETI: effector-triggered immunity
HR: hypersensitive response
TMV: Tobacco Mosaic Virus
ROS: reactive oxygen species
NBS: nucleotide-binding site
LRR: leucine-rich repeat
Y2H: yeast-two-hybrid
HMM: Hidden Markov Model
