# Supplementary figures and images for: A novel hairpin library-based approach to identify NBS–LRR genes required for effector-triggered hypersensitive response in Nicotiana benthamiana
Source: Plant Methods. 2017 Apr 28;13:32. doi: 10.1186/s13007-017-0181-7 (PMC5408436; doi:10.1186/s13007-017-0181-7)

A

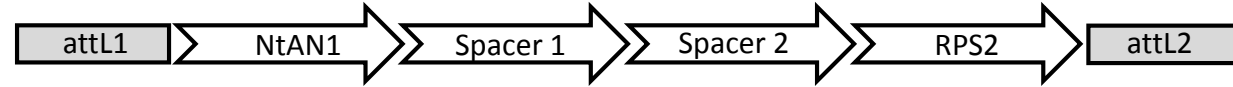

B

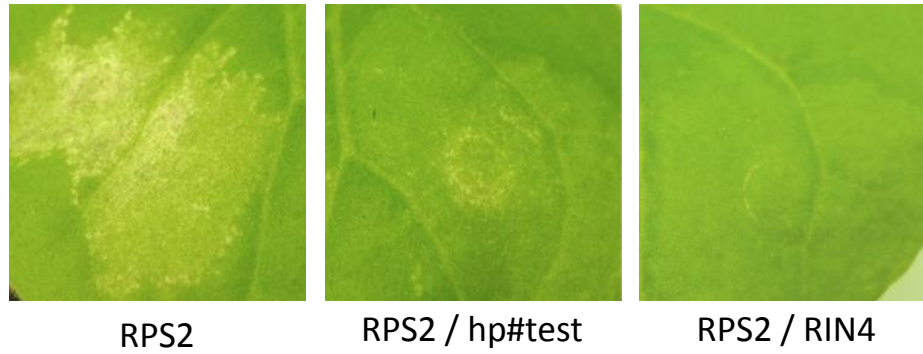

C

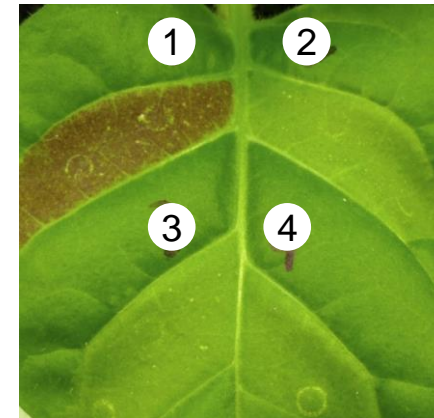

- 1 AcMYB110 / empty vector
- 2 AcMYB110 / hp#test
- 3 AcMYB110 / hp#NtAN1
- 4 hp#test / empty vector

Supplement: Supplementary file 1 — Additional file 1: Figure S1. Multiple gene silencing proof-of-concept assay. A construct including a fragment of NtAN1, two DNA spacer sequences and a fragment of RPS2 (each of 150 bp) and flanked by the Gateway attL1 and attL2 borders (A) was synthesized and recombined by LR reaction into the pTKO2 vector to produce the pTKO2-hp#test construct. (B) Agrobacterium-mediated transient expression of RPS2 triggers an HR in N. benthamiana leaf and RIN4 negatively regulates RPS2 activation [37]. Here we show that when RPS2 and the hp#test construct are co-infiltrated (ratio 1:1) the HR is severely reduced, demonstrating that the 150 bp-kmer at the last position of the construct efficiently mediates RPS2 silencing. (C) AcMYB110 is a positive regulator of the anthocyanin biosynthetic pathway in kiwifruit. It was shown previously that AcMYB110 requires the endogenous NtAN1 bHLH transcription factor to induce anthocyanin biosynthesis when expressed in tobacco leaf [38]. Here we show that when AcMYB110 and either the hp#test construct (2) or a specific NtAN1 hairpin construct (3) are co-infiltrated (ratio 1:1), no anthocyanin accumulates in the leaf demonstrating that in both cases the endogenous NtAN1 is efficiently silenced. [file 13007_2017_181_MOESM1_ESM.pdf]
